# Acetylation of proximal cysteine-lysine pairs by alcohol metabolism

**DOI:** 10.1016/j.redox.2024.103462

**Published:** 2024-12-12

**Authors:** Courtney D. McGinnis, Peter S. Harris, Brenton I.M. Graham, John O. Marentette, Cole R. Michel, Laura M. Saba, Richard Reisdorph, James R. Roede, Kristofer S. Fritz

**Affiliations:** Graduate Program in Toxicology, Skaggs School of Pharmacy and Pharmaceutical Sciences, University of Colorado Anschutz Medical Campus, Aurora, CO, USA

**Keywords:** Cysteine proteomics, Redox, Acetylation, Protein modeling, Alcohol-associated liver disease, Mass spectrometry

## Abstract

Alcohol consumption induces hepatocyte damage through complex processes involving oxidative stress and disrupted metabolism. These factors alter proteomic and epigenetic marks, including alcohol-induced protein acetylation, which is a key post-translational modification (PTM) that regulates hepatic metabolism and is associated with the pathogenesis of alcohol-associated liver disease (ALD). Recent evidence suggests lysine acetylation occurs when a proximal cysteine residue is within ∼15 Å of a lysine residue, referred to as a cysteine-lysine (Cys-Lys) pair. Here, acetylation can occur through the transfer of an acetyl moiety via an S → N transfer reaction. Alcohol-mediated redox stress is known to occur coincidentally with lysine acetylation, yet the biochemical mechanisms related to cysteine and lysine crosstalk within ALD remain unexplored. A murine model of ALD was employed to quantify hepatic cysteine redox changes and lysine acetylation, revealing that alcohol metabolism significantly reduced the cysteine thiol proteome and increased protein acetylation. Interrogating both cysteine redox and lysine acetylation datasets, 1280 protein structures generated by AlphaFold2 represented by a 3D spatial matrix were used to quantify the distances between 557,815 cysteine and lysine residues. Our analysis revealed that alcohol metabolism induces redox changes and acetylation selectively on proximal Cys-Lys pairs with an odds ratio of 1.88 (p < 0.0001). Key Cys-Lys redox signaling hubs were impacted in metabolic pathways associated with ALD, including lipid metabolism and the electron transport chain. Proximal Cys-Lys pairs exist as sets with four major motifs represented by the number of Cys and Lys residues that are pairing (Cys_1_:Lys_1_, Cys_x_:Lys_1_, Cys_1_:Lys_x_ and Cys_x_:Lys_x_) each with a unique microenvironment. The motifs are composed of functionally relevant Cys-Ly altered within ALD, identifying potential therapeutic targets. Furthermore, these unique Cys-Lys redox signatures are translationally relevant as revealed by orthologous comparison with severe alcohol-associated hepatitis (SAH) explants, revealing numerous pathogenic thiol redox signals in these patients.

## Graphical abstract

**Figure:**
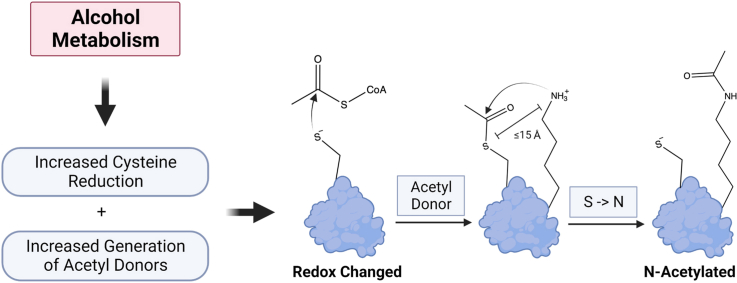

## 1 Introduction

Alcohol is a toxic and habit-forming substance that is consumed by over 62 % of people in the United States [1]. Worldwide, chronic consumption of alcohol contributes to disease etiology in approximately 46 % of patients with liver disease and alcohol use is among the leading causes of preventable death [2]. Unfortunately, aside from abstinence and liver transplantation there are no approved therapeutic interventions [3]. The challenges impeding the development of therapeutics for alcohol-associated liver disease (ALD) are multifaceted, encompassing disease heterogeneity among individuals with ALD adding to the complexity of clinical trials, late stage diagnosis, and limited biochemical understanding of early stages of ALD progression [4]. Further characterization of biochemical changes driven by alcohol metabolism will provide therapeutic advances ameliorating hepatic alcohol toxicity.

The pathological onset and development of ALD includes the disruption of many biochemical processes resulting in metabolic, genetic, and proteomic alterations [3]. One such result attributed to metabolic disruption, is the robust increase in the post-translational modification (PTM), lysine (Lys) acetylation, driven by alcohol metabolism [[5], [6], [7]]. Like many other PTMs, Lys acetylation is critical for regulating a host of metabolic and cellular functions, such as cellular respiration, fatty acid handling and signaling [8]. The increase of Lys acetylation has been well characterized within ALD models and is associated with metabolic disruption [9,10]. Furthermore, a recent thiol-centric proteomic analysis in ALD revealed the global increase of hepatic protein cysteine (Cys) residues in their thiol/thiolate form (reduced) [11]. This study mapped fundamental protein thiols and identified locations of redox perturbation attributed to alcohol metabolism which can be used to extrapolate the response of redox regulated enzymes. The observed Cys reduction confirms that the hepatic proteome responds to alcohol metabolism through multiple routes of PTM signaling. One unique mechanism of modification is the non-enzymatic transfer of an acetyl moiety from a Cys thiol to a Lys ε-amine [12]. Our recent findings of increased Lys acetylation and a reduced thiol proteome suggests cysteine-lysine (Cys-Lys) proximity may be a key metabolically derived feedback loop regarding Cys redox changes and Lys acetylation in response to alcohol toxicity [10,11].

Emerging research suggests Lys acetylation can be facilitated by the proximity of an acetylated Cys thiol [13]. More specifically, Lys acetylation was found to be significantly increased when a Lys is within 15 Å of a Cys, termed a proximal Cys [12,13]. Here, the mechanism of acetylation is proposed to occur due to the Cys thiol attacking the thiol ester carbonyl of the acetyl moiety of acetyl coenzyme A (Ac-CoA), resulting in an S-acetylation intermediate followed by S → N transfer of the acetyl moiety to a nearby Lys ε-amine [12]. Due to pK_a_ ∼8.3–8.5 Cys thiol residues more readily react with Ac-CoA molecules; thus, stabilizing the acetyl moiety and enabling the Lys ε-amine to react with the thiol ester carbonyl rather than Ac-CoA(12). This concept has been further supported by the *in vitro* identification of the proximity of nucleotide binding sites increasing Lys acetylation [14]. Further, the structural similarities between co-factors enables association with similar binding sites resulting in non-discriminate CoA binding and enabling acyl modifications on Lys proximal to that binding pocket [12,13,15]. Here, we are structurally focused on alcohol-related changes to Cys and Lys residues by evaluating the distance between the thiol sulfur atom (S^−^) and the ε-amine nitrogen (NH_3_^+^) atom across three-dimensional (3D) protein structures, while noting co-factor binding affiliation. Consequently, uncovering Cys-Lys residues significantly impacted by alcohol metabolism expands the scope of enzymes critical to ALD pathology.

Here, we present the first analysis integrating hepatic quantitative Cys redox proteomics and Lys acetylation to experimentally determine proximal Cys-Lys pairs. Evaluating Cys-Lys relationships within a model of ethanol consumption is critical to further understanding hepatic alcohol-induced biochemical changes and the interplay between PTMs and protein function. The implementation of a 6-week Lieber-DeCarli model recapitulates early stages of ALD pathology and enables the examination of proteomic changes occurring during alcohol exposure and the onset of ALD(7). The integrated evaluation of Cys redox and acetyl-Lys proteomic datasets was coupled with cutting-edge predictive protein structure modeling which revealed novel spatial interactions of Cys-Lys amino acids and their modifications. StructureMap enabled the application of structural data from the AlphaFold2 artificial intelligence program [16,17]. The integration of quantitative proteomic data and structural information revealed an increase in the likelihood of a change in Cys redox status when Cys and Lys residues are within 15 Å. Furthermore, this study identified a trend among these proximal Cys-Lys pairs where cofactor binding, such as metal or NAD^+^, is associated with modifications [14,15]. Finally, database mining across a cohort of severe alcohol-associated hepatitis (SAH) patients revealed numerous orthologous proteins containing regulatory Cys-Lys pairs. Supporting that specific translational Cys-Lys pairs may be key therapeutic targets for the treatment of end-stage liver disease [18].

## 2 Materials and methods

### 2.1 Animals

All animal-related experiments were approved by the Institutional Animal Care and Use Committee of the University of Colorado and were performed in accordance with published National Institutes of Health guidelines. Healthy male C57BL/6J mice were purchased from The Jackson Laboratory (Bar Harbor, ME) and housed in a temperature-controlled (20–22 °C) room on a 6am to 6pm light/dark cycle. 12-week-old mice were fed a modified Lieber-DeCarli liquid based-diet (Bio-Serv, Frenchtown, NJ) for 6 weeks. The diet consisted of 44 % fat-derived calories, 16 % protein-derived calories and the remaining balance was comprised of either carbohydrate or alcohol-derived calories. Control (n = 5) and alcohol (n = 5) mice were pair-fed where alcohol-derived calories were replaced with calories from the carbohydrate source (maltose-dextrin). Mice in the alcohol group began the study at 2 % alcohol (v/v) and the concentration of alcohol was increased every week until 6 % alcohol (v/v) for the final week as previously described [11].

### 2.2 Cysteine proteomic analysis

The click chemistry Cys redox assay used was adapted from Yang et al. [19] and Pertova et al. [20] to quantify protein Cys residues that underwent a redox change due to alcohol metabolism by nHPLC-MS/MS. This assay utilized an isotopically labeled biotin-azide to “click” the free protein thiols of reduced Cys modified by alkynyl iodoacetamide. The pair-fed control and alcohol-fed murine hepatic peptides were combined 1:1 and were used to generate an accurate mass and retention time (AMRT) library [11]. General protein quantitation (GPQ) was performed on the individual samples and used to normalize Cys redox proteomic abundance changes. MS quantitation data was extracted and aligned for both the protein quantitation and the Cys proteomic analysis as described elsewhere [11].

### 2.3 Acetylomic sample preparation

A different piece of hepatic tissue from the same mice assayed in the click chemistry Cys redox experiment (n = 5, per group) was homogenized by bead milling using a Qiagen TissueLyser LT set to 50Hz for 6 min. Samples were homogenized in a 10:1 diluted urea lysis buffer as outlined in the PTMScan HS Acetyl-Lysine Motif kit (Ac–K kit) (Cell Signaling Technology #46784) at a ratio of 10 μL/mg of tissue (Danvers, MA). Samples were spun down in a microcentrifuge at room temperature to pellet any remaining tissue debris not solubilized and then a BCA protein assay was performed on the supernatant. Equivalent 4 mg aliquots of protein were taken from each sample. One hundred nanograms of Acetyl BSA internal standard was added to each sample followed by reduction, alkylation, and digestion with trypsin at 1:60 enzyme:protein for 18 h. After digestion, samples were desalted on C_18_ SPE columns as previously described [7,10]. The peptide eluent was divided to yield four 1 mg aliquots and dried down in 2 mL microcentrifuge tubes using a Savant SPD1010 SpeedVac Vacuum Concentrator under reduced pressure. One aliquot was used for general protein quantitation and another aliquot was used for Immunoprecipitation (IP) of acetyl-Lys peptides. The IP and subsequent desalting of Immunoprecipitated peptides using Piece™ C18 Spin Tips (Thermo Scientific, PN 84850) was performed exactly as stated in steps III and IV of the protocol provided with the Ac–K kit. Acetyl peptides were dried down via SpeedVac and stored at −80 °C for LC-MS/MS analysis (Waltham, MA).

### 2.4 General protein quantitation

GPQ sample aliquots from the acetylomic sample preparation were suspended in a 3 % acetonitrile with 0.1 % formic acid solution containing 15 fmol/μL of Procal Spike Mix Retention Time Standards (TRK-1-10pmol) to control for instrument variability [21]. Samples were then loaded onto a 2 cm PepMap 100, nanoviper trapping column and chromatographically resolved on-line using a 0.075 × 150 mm, 3.0 μm EASY-Spray PepMap RSLC C_18_ reverse phase nano column and an Ultimate 3000 RSCLnano LC system (Thermo Scientific, Waltham, MA). Mobile phases consisted of water +0.1 % formic acid (A) and 100 % acetonitrile + 0.1 % formic acid (B). Samples were loaded onto the trapping column at 5.0 μL/min for 4.5 min at initial condition before being chromatographically separated at a flow rate of 300 nL/min using a gradient of 3–7% B over 2.5 min, 7–33 % B over 93 min for a total 95.5 min gradient at 40 °C. The gradient method was followed by a column wash at 70 % B for 5 min. Data was collected on an Orbitrap Eclipse (Thermo Scientific) equipped with an EASY-Spray Source operated using intensity dependent Collision induced dissociation (CID) MS/MS to generate peptide ID's. MS^1^ spectra were acquired in the Orbitrap (Resolution = 120k; AGQ target = 100 %; MaxIT = Auto; RF Lens = 30 %; mass range = 350–1500; Profile data) (Waltham, MA). Precursors selected for MS/MS were filtered by mono isotopic precursor selection (MIPS) model set to peptide with an intensity threshold of 15000 and only charge states 2–6 were allowed. Dynamic exclusion was employed for 10 s and the dependent scan for a single charge state per precursor only was set to true. MS^2^ spectra were collected using CID in the linear ion trap with a cycle time of 1 s between MS Orbitrap scans (Isolation window = 1.2 m/z [quadrupole], rate = rapid; AGQ target = Standard; MaxIT = Dynamic; CID = 35 %).

Data were searched and extracted using SEQUEST HT and the label-free quantitation workflow in Proteome Discover (PD) software version 2.5.0.400 that includes the minora feature detector, feature mapper, and precursor ions quantifier algorithms. Spectra were searched against a combined Procal Retention Time (RT) standard peptide database and the SwissProt *Mus musculus* database downloaded July 17th, 2023, allowing up to 2 missed tryptic cleavages with fixed carbamidomethylation (cysteine) and dynamic deamidation (asparagine or glutamine) and oxidation (methionine) modifications. The monoisotopic peptide mass tolerance allowed was ±10.0 ppm and the MS/MS tolerance was ±0.5Da. P-values related to peptide-spectrum matches were adjusted to a 1 % false discovery rate (FDR) using the percolator algorithm. Additionally, only high confidence peptides were allowed for protein scoring and a protein FDR cut-off of 1 % was also used. A signal to noise (S/N) threshold of 1.5 was set for minora feature detector and at least 6 points had to be found across a peak. Peptide spectrum matches (PSMs) confidence levels were set to high for feature ID linking. A coarse retention time (RT) alignment of data was performed using vendor suggested default values. Following coarse RT alignment, the retention time tolerance was automatically determined by PD software based on coarse aligned data files, mass tolerance of 10 ppm, minimum S/N threshold of 5 were set for feature linking and mapping. Precursor quantification was based on area and protein abundances were estimated by summing the abundances of all peptides matched to a specific protein using the Unique + Razor setting in PD software. The total Procal summed abundance for each sample was used to adjust protein abundances of all other identified and quantified proteins in that sample to control for instrument variability. The GPQ Procal corrected protein abundances were then used to adjust acetyl peptide abundance on a per protein and sample basis.

### 2.5 Acetylomic analysis by LC-MS/MS

Dried immunoprecipitated peptides from the acetylomic sample preparation were re-suspended in 3 % acetonitrile 0.1 % formic acid solution with 15 fmol/μL of Procal Spike Mix. LC-MS/MS data was acquired on one third of each acetyl sample using the same method parameters as the GPQ data acquisition above with the following exceptions: a shorter gradient was used for peptide separation, the ion trap scan rate was set to Normal, and maximum injection time type was set to Auto. The shorter gradient used is described as follows: Samples were loaded onto the trapping column at 5.0 μL/min for 5 min at initial condition before being chromatographically separated at a flow rate of 300 nL/min using a gradient of 3–7% B over 3 min, 7–26 % B over 40 min, and 26–33 % B over 4 min for a total 47 min gradient at 40 °C. The gradient method was followed by a column wash at 70 % B for 4 min.

Data were searched and extracted using the same parameters as GPQ data with the following exceptions: Up to 4 missed tryptic cleavages were allowed, acetylation (lysine) was an allowed dynamic modification, and spectra were searched against a combined Procal RT standard peptide database, SwissProt *Mus musculus* database downloaded July 17th, 2023, and bovine serum albumin (BSA) database. Protein level summed area abundances of Procal peptides and spiked in acetylated BSA peptides were used to correct all identified and quantified acetyl peptide area abundances on a sample per sample basis. First, the total sum of Procal area abundance within each sample was used to correct the total sum of spiked acetylated BSA peptide abundances within each sample. A normalization factor was then generated for each sample using the total Procal adjusted acetylated BSA signal found within each sample and these factors were used to normalize acetyl peptide levels within each sample. The Procal adjusted acetylated BSA normalized acetyl peptide abundance data was then normalized on a protein/sample specific basis based on Procal adjusted general protein quantitation data abundances. GPQ sample E5 was used as the reference data file for GPQ factors for each protein. To summarize, Procal standards were used to control for run-to-run variability, acetyl BSA was used to control for sample preparation variation, and general protein quantitative data was used to adjust for protein abundance differences between each sample.

Statistical analysis was then performed in Mass Profiler Professional software V.15.1 (Agilent Technologies) on adjusted acetyl peptide data using an unpaired *t*-test with Benjamini-Hochberg multiple testing correction (Santa Clara, CA). A corrected p-value (p (Corr)) ≤ 0.05 and log_2_ fold change (FC) cut-off of |0.58| were used to determine statistical significance of acetylated peptides [22].

### 2.6 Cysteine-lysine proximity and structural analysis

1280 unique UniProt IDs were used to extract CIF (crystallographic information files) and PAE (predicted aligned error) files from the AlphaFold Protein Structure Database version 4 (AlphaFold DB accessed 03.09.2024; http://alphafold.ebi.ac.uk) [17]. The site-directed PTM proximity analysis evaluated the distance between amino acid residues experimentally determined to be acetylated or redox changed with predicted proteins structures using Python Program StructureMap described in Bludau et al. (http://github.com/MannLabs/structuremap) [16]. Peptides with multiple modified sites were stratified into individual sites, and the FC values were averaged. The lowest corrected p-value associated with each individual site was used for subsequent analysis. The Lys amine and the Cys thiol were assigned coordinates on experimentally pertinent proteins then the proximity of all corresponding Lys and Cys residues was quantified to identify all possible Cys-Lys pairs (https://github.com/FritzLaboratory). Amino acid residues with a quality score below 70 % were excluded according to AlphaFold's confidence metrics. Additionally, amino acid residues with a PAE greater than 15 Å were excluded from this analysis because 15 Å is predicted to be necessary for biological interactions [13]. As a novel technology AlphaFold2 has several limitations due to current modeling capacity. For example, in this analysis, 20 proteins from our datasets were unable to be predicted and/or represented by a distance matrix by AlphaFold2. Additionally, only 70 % of residues had locations predicted with sufficient accuracy to be included in this analysis. The ability to accurately predict protein structure by AlphaFold2 is dependent on reliable homology protein sequences and preexisting structures suggesting that less studied proteins are more likely to be excluded from this analysis. Furthermore, the structures present in the AlphaFold DB are limited to their monomeric form therefore we were unable to analyze potential Cys-Lys pairs between multimeric proteoforms [17,23].

The 3D structure visualization of AlphaFold2 DB predicted structures was conducted in Discovery Studio 2021 (BIOVIA). AlphaFold2 DB protein structures visualized in this manuscript include: glutaredoxin-related protein 5 (Glrx5) (AF-Q80Y14–F1), enoyl-CoA hydratase 1 (Echs1) (AF-Q8BH95–F1), peroxisomal 2,4-dienoyl-CoA reductase (Decr2) (AF-Q9WV68–F1) and alcohol dehydrogenase (Adh1) (AF-P00329-F1).

### 2.7 Pathway analysis of acetylated and redox-modified protein

Peptides identified by the acetylomic analysis were condensed to the protein level by combining peptide abundance values and the inverse fold change was used due to the common association of acetylation as an inhibitory modification [24]. Additionally, peptides from the Cys redox proteomic analysis were condensed to the protein level by combining modified peptide abundance values. All proteomic analyses were performed on the same murine liver samples. Ingenuity Pathway Analysis (IPA) Software (QIAGEN) and protein IDs were used to identify associated canonical pathways and toxicity phenotypes. Expression analysis with a right-tailed Fisher's exact test with Benjamini-Hochberg multiple testing correction was used to identify significantly enriched pathways. A corrected p-value of <0.05 was considered statistically significant. Activation and inhibition z-scores were calculated by IPA's z-score algorithm to predict significant canonical pathway activation (z-score>2) or inhibition (z-score < −2). The UniProt application programming interface (API) (version 2024_03) was queried to identify the subcellular location and molecular function annotation for all proteins found in both the acetylomic and redox proteomic datasets (see Supplemental 1 Cys Lys StructureMap). Proteins associated with one or more subcellular locations or functions were summarized for all possibilities. The motif enrichment analysis displayed in Table 1 was conducted with Database for Annotation, Visualization and Integrated Discovery (DAVID) (see Supplemental 3 CysLys Motif). CysLys orthologous subcellular location, canonical pathway enrichment, and metabolic pathways were identified by DAVID and substrate usage were determined by Reactome (https://reactome.org/) [25,26].

**Table 1:** Description of Cys-Lys pair motif and associated subcellular compartments, biological processes, enzymatic class, and the number of Cys-Lys pairs in each motif.

	Cys_1_:Lys_1_	Cys_x_:Lys_1_	Cys_1_:Lys_x_	Cys_x_:Lys_x_
Subcellular location	Mitochondria	Mitochondria	Cytoplasm	Mitochondria
Biological process	Metabolic pathways	Metabolic pathways	Coronavirus disease - COVID-19	Carbon metabolism
Enzymatic class	Oxidoreductase	Oxidoreductase, Metal Ion Binding	Protein binding, nucleotide binding	Metal Ion Binding
# of Cys-Lys Pairs	345	178	419	23

### 2.8 Human ortholog identification

Murine proteins containing Cys-Lys pairs with significantly modified residues were cross-referenced with a previously published human SAH explant acetylomic dataset [27]. The BioMart R package was implemented to access Ensembl 111: Jan 2024 (https://www.ensembl.org) [28]. UniProt version 2024_03 was used to align ADH1 amino acid sequences (https://www.uniprot.org/) [29].

### 2.9 Immunoblotting

30 μg of murine hepatic whole tissue lysate was prepared by adding 4x Laemmli loading buffer (Bio Rad) then denatured by boiling sample for 5 min at 92 °C. Protein was separated by sodium dodecyl sulfate polyacrylamide gel electrophoresis (Waltham, MA). Total protein abundance was determined using 2,2,2-trichloroethanol (Sigma-Aldrich) and used to normalize the signal from specific antibodies (St. Louis, MO). The anti-acetylated-lysine antibody was purchased from Cell Signaling Technology (#9441) and was used at a 1:1000 dilution (Danvers, MA). Blots were visualized using a Bio-Rad ChemiDoc MP Imaging Systems and analyzed using Bio-Rad ImageLab 6.0.1 software. For immunoblot densitometry, graphs represent the average within each group (n = 5, per group) with error bars representing the within group standard deviation.

### 2.10 Statistical analysis

Proteins were divided into four mutually exclusive groups 1) proteins with at least one peptide that contained significantly (p (Corr)≤0.05, log_2_FC≥|0.58|) acetylated/deacetylated Lys and at least one peptide having Cys redox significantly (p (Corr)≤0.05, log_2_FC≥|0.58|) changed, 2) proteins containing a peptide with a significantly acetylated/deacetylated Lys and no peptides with a significantly changed Cys, 3) proteins with a peptide containing a significantly changed Cys, and 4) proteins which have no significantly modified peptides. At the protein level, a Fisher's Exact test was used to determine if more co-occurring alcohol-induced peptide modifications on the same protein (Supplemental Tables 1 and 2) were observed than were expected by chance. For all Cys-Lys pairs within 15 Å of each other, a Fisher's Exact test and odds ratio (OR) were used to compare the odds of Lys acetylation between pairs with a modified Cys and pairs without a modified Cys (Supplemental Table 3). For all the significant PTM (acetylated Lys and modified Cys), distances to the closest proximal Cys (for Acetyl-Lys) and Lys (for Cys Mod) residue were also examined (Fig. 4C). For immunoblot densitometry, graphs represent the within treatment group average (n = 5, per group) with error bars representing the standard error of the mean (SEM). Statistical analyses and graphical representations were completed and generated by GraphPad Prism (10.1.0) and R Statistical Software (9.0)/RStudio 4.3.3 (2023.12.1 + 402).

## 3 Results

### 3.1 Characterization of alcohol-induced hepatic acetylome modifications

**Fig. 1:**
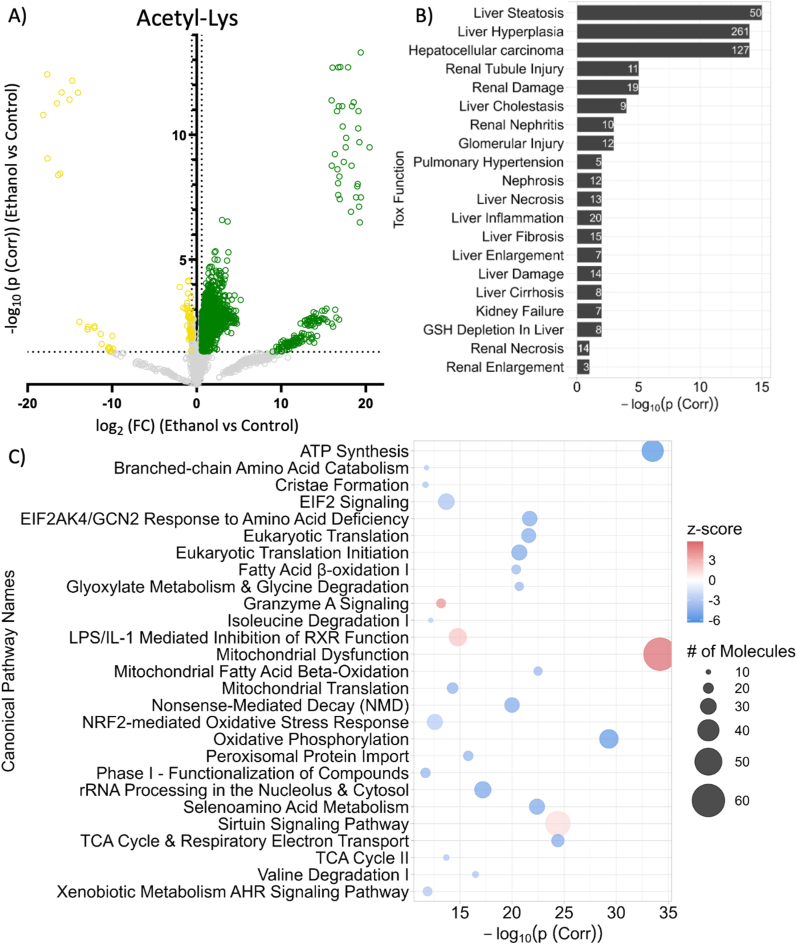
Characterization of alcohol-induced hepatic acetylome modifications. A) This volcano plot depicts acetylated (dark green log_2_(FC)≥|0.58|, -log_10_(p (Corr))>1.30) or deacetylated (dark yellow -log_2_FC≤|0.58|, -log_10_(p (Corr))≥1.30) peptides due to alcohol exposure. Non-significant peptides that are below the FC threshold are shown in grey. The dashed horizontal line marks the significance threshold (p (Corr) = -log_10_(0.05)) and vertical marks indicate log_2_(FC) of |1.5| and -|1.5|. B) Bar plot of whole liver acetylomic pathway enrichment of toxicity endpoints with –log_10_(p (Corr)) representing enrichment score and values within each bar indicating the number of molecules identified within each pathway. C) Bubble plot of acetylomic canonical pathway enrichment with the -log_10_(p (Corr)) displaying enrichment score, number of molecules denoted by size, and color representing pathway activation (orange) and inhibition (purple). ALT: Alanine aminotransferase; ATP: Adenosine triphosphate; EIEF2: Eukaryotic initiation factor 2; GCN2: Serine/threonine-protein kinase general control nonderepressible 2; TCA: Tricarboxylic acid cycle.

**Fig. 2:**
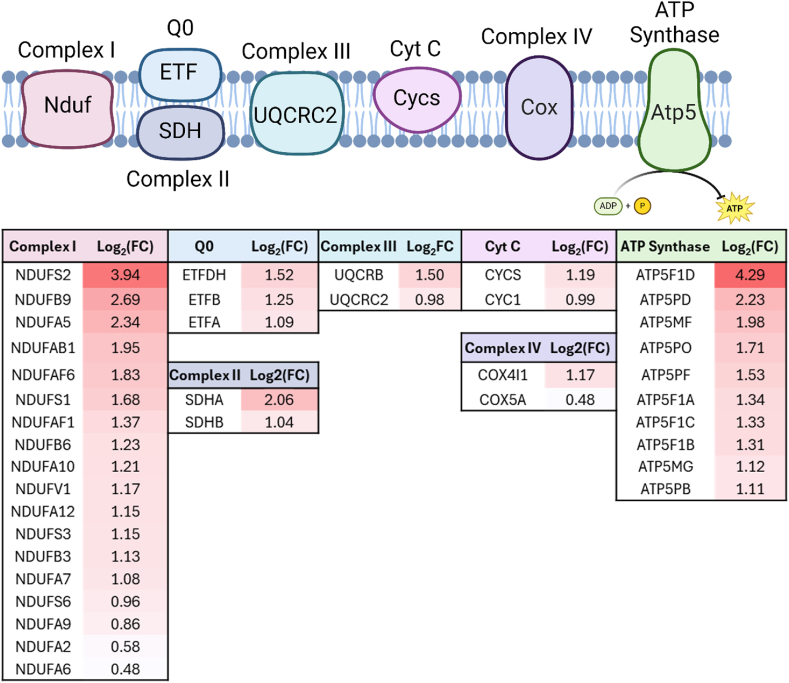
Alcohol induced acetylation of proteins within the electron transport chain. Illustration of proteins containing acetylated lysines significantly changed by multiple comparison-corrected test within the electron transport chain. The data is presented as log_2_ fold-change of ethanol over control. Figure made in BioRender.com. Atp5: Adenosine triphosphate synthase 5; Cox; Cyclooxygenase, Cyc: Cytochrome subunit; Cyt C; Cytochrome complex, ETF; Electron-transfer flavoprotein, Nduf; NADH:ubiquinone oxidoreductase, SDH; Succinate dehydrogenase complex.

The analysis of whole liver acetylomic profiles revealed significant differences in murine hepatic protein acetylation due to alcohol metabolism. A total of 2266 peptides from 1006 proteins were found to be acetylated at one or more Lys sites. Of those, 1373 peptides were significantly acetylated in response to chronic alcohol consumption and contained an acetylated Lys with a log_2_ difference greater than 0.58 (i.e., 50 % higher acetylation in the alcohol group compared to the control group) (dark green Fig. 1A). In contrast, 73 peptides were found to be significantly deacetylated with chronic alcohol consumption (dark yellow Fig. 1A). Additionally, 120 peptides were found to have increased acetylation by 50 % or more (log_2_(FC)≥0.58 difference) and 38 were found to have decreased acetylation (log_2_(FC) ≤ -0.58 difference) but were not significantly changed by the multiple comparison-corrected threshold (p (Corr)≤0.05) (light green and light yellow, respectively Fig. 1A). The phenomenon of more acetylation than deacetylation due to alcohol in the acetylomics was validated by immunoblot, observing a 1.92-fold increase in whole cell liver lysates (Supplemental Fig. 1). The IPA Toxicity Function analysis of proteins found to be acetylated or deacetylated examined associations with pathological endpoints and identified diseases linked with chronic alcohol overconsumption, such as liver steatosis and hepatocellular carcinoma (Fig. 1B) (Supplemental 2 IPA Acetylome and CysLys). The canonical pathway with the highest enrichment score (i.e., largest -log_10_(p (Corr)) was mitochondrial dysfunction which is associated with the development of hepatic lipid accumulation (Fig. 1C) [6]. Proteins driving this association were primarily components of the electron transport chain (ETC) and are involved in ATP synthesis, the second most enriched pathway. Proteins driving the enrichment included multiple subunits from Complex I, Complex II, Complex III, Cytochrome C, Complex IV, and ATP synthase (Fig. 2). Eighteen subunits of Complex I were found to be acetylated with NDUFS2 having the largest fold change, indicating key acetyl-mitochondrial disruptions in energy homeostasis through the electron transport chain during alcohol toxicity (Fig. 2).

### 3.2 Integration of alcohol-induced cysteine redox and lysine acetylation proteomic datasets

**Fig. 3:**
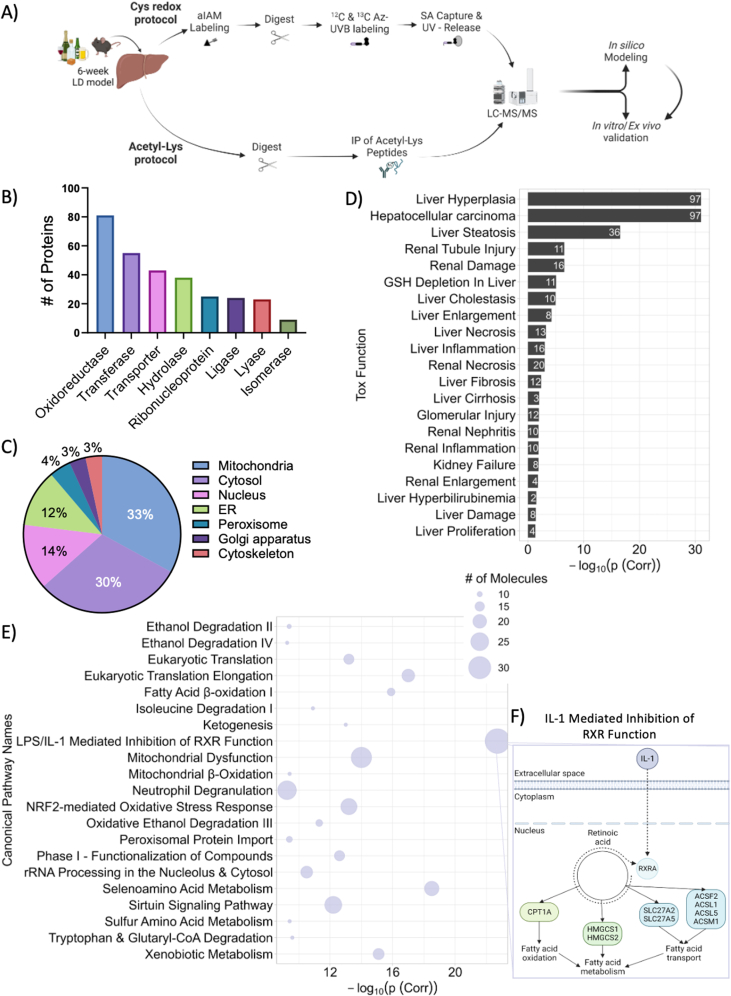
Pathway enrichment analysis of proteins identified by the cysteine proteomic and acetylomic analysis. A) Schematic for the development and integration of a cysteine proteomic and acetylomic analysis on 6-week Lieber-DeCarli (LD) mouse tissue. B) Bar plot depicts the number of acetylated and redox changed hepatic proteins within each enzymatic class. C) Pie chart displaying the percentage of acetylated and redox changed hepatic proteins within each subcellular compartment. D) Bar plot of selected toxicity functions identified through of analysis of acetylated and redox modifies hepatic proteins with –log_10_(p (Corr)) representing enrichment score and values within each bar indicating the number of molecules identified within each pathway. E) Canonical pathway enrichment of acetylated and redox modified hepatic proteins with the -log_10_(p (Corr)) representing enrichment score and the number of molecules represented by size. F) IL-1 mediated inhibition of RXR function pop-out depicts the fatty acid metabolizing enzymes regulated by RXRA (enrichment by IPA). Figure made in BioRender.com. ACS: Acetyl-CoA synthetase; aIAM: Alkynyl iodoacetamide; Az-UVB: UV cleavable biotin-azide; CPT: Carnitine palmitoyltransferase; ER: Endoplasmic reticulum; HMG: Hydroxymethylglutaryl-CoA synthase; IL-1: Interleukin 1; IP: Immunoprecipitation; LC-MS/MS: Liquid Chromatography tandem mass spectrometry; LD: Lieber-DeCarli; RXRA: Retinoid X receptor alpha; SLC: Solute carrier; SA: Streptavidin.

Alcohol-induced changes in murine hepatic thiol redox proteome were evaluated previously, noting that the majority of Cys thiols were significantly reduced [11,15]. The whole-liver acetylomic analysis described above (Fig. 1, Fig. 2) was conducted on the same biological replicates used in the Cys proteomic analysis for PTM proteomic integration (Fig. 3A). Proteins that underwent an alcohol-induced Lys acetylation were more likely to have a change in Cys redox status than proteins that did not have alcohol-induced Lys acetylation (OR = 1.47, p-value = 0.034, Supplemental 1 Cys Lys StructureMap). Protein with an alcohol-induced Cys redox change had a higher probability of having alcohol-induced Lys acetylation than proteins without an alcohol-induced redox change (OR = 1.33, p-value = 0.054, Supplemental 2 IPA Acetylome and CysLys). Proteins identified within both datasets were characterized by querying UniProt API to identify the molecular functions enriched. Oxidoreductase enzymes or redox reactive proteins contained the greatest number of proteins (Fig. 3B). Furthermore, UniProt API was queried to categorize the subcellular localization of these proteins (Fig. 3C). Mitochondrial and cytosolic proteins were the first and second most common locations of enzymes found in both the Cys redox and acetyl-Lys integration analysis. Next, Tox Functions were identified using IPA for proteins containing both Cys redox changes and Lys acetylation. The greatest enrichment for hepatic disease pathologies were liver hyperplasia, hepatocellular carcinoma, liver steatosis, and glutathione depletion (Fig. 3D). IPA canonical pathway analysis identified enrichment of pathways related to the development of liver disease, including sirtuin (Sirt) signaling, mitochondrial dysfunction, and xenobiotic metabolism (Fig. 3E). Furthermore, the pathway with the highest enrichment was lipopolysaccharide/Interleukin 1 (LPS/IL-1) mediated inhibition of RXR function with direct implications for fatty acid metabolism and hepatic steatosis (Fig. 3F).

**Fig. 4:**
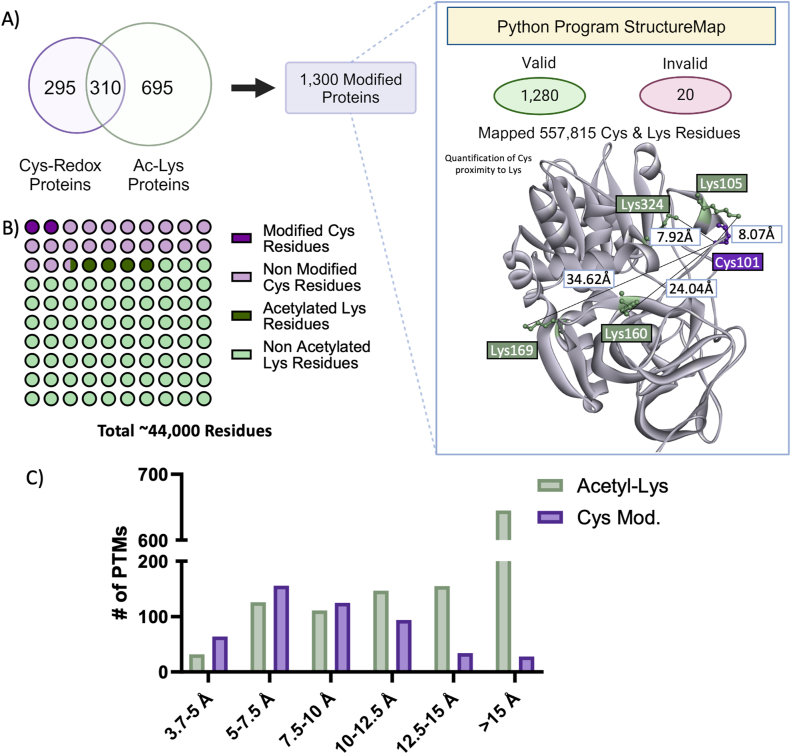
StructureMap was used to determine Cys and Lys proximity of AlphaFold2 predicted protein structures found to be modified by alcohol. A) Proteins found to be modified by alcohol metabolism were used in the Python program StructureMap to evaluate the distance between Cys and Lys residues on predicted protein structures. The program mapped 1281 of the 1300 proteins input and quantified the distance between 557,815 Cys-Lys pairs. Adh1 (AF-P00329-F1) demonstrated the use of AphaFold2 predicted structure to map amino acid residues. B) Breakdown of Cys residues and Lys residues within the proteins mapped by StructureMap either modified or unmodified containing circles representative of approximately 450 amino acids. C) Bar plot depicting number of Cys residues with a redox change and distance to nearest Lys residue and acetylated Lys residue and the distance to nearest Cys residue. Acetyl-Lys: Acetylated Lysine; Adh1: Alcohol dehydrogenase; Cys-Redox: Cysteine redox proteome.

### 3.3 Chronic alcohol consumption modifies cysteine-lysine pairs

StructureMap enabled the integration of Cys proteomic and Lys acetylomic data with AlphaFold2's structural information. AlphaFold2 is an artificial intelligence system able to predict 3D protein structure [17]. A total of 1300 unique proteins identified within our Cys proteome and acetylome datasets were uploaded to StructureMap for the quantification of distance between Cys and Lys residues on fully folded structures plotted by distance matrix (Fig. 4A–Supplemental 1 Cys Lys StructureMap). Of the 1300 proteins, StructureMap was able to confidently map 1280 proteins. In total, the distances between 557,815 Cys and Lys residues (Cys-Lys pairs) were analyzed. These Cys-Lys pairs include 9895 unique Cys residues and 33,849 unique Lys residues (Fig. 4B). Following the implementation of AlphaFold2's confidence metrics with a cut-off of 70 % quality score and 15 Å predicted aligned error (PAE), we identified 388,698 Cys-Lys pairs that we used to evaluate the relationship between the physical distance between Cys-Lys residues and the presence of alcohol-induced PTMs of these residues. Of these Cys-Lys pairs, 39,696 have at least one modified residue within the pair, 25,886 pairs have at least one redox changed Cys, and 15,724 pairs have an acetylated or deacetylated Lys residue. When only considering Cys-Lys pairs within 15 Å (28,238 pairs), the probability that the Lys is acetylated is higher among pairs with a modified Cys than the pairs with no modified Cys (OR = 1.89, p-value<0.001) (Supplemental 3 CysLys Motif). Here in Fig. 4C the number of modified Cys residues to the nearest Lys residue and acetylated Lys residues to the nearest Cys residue was stratified by distance to inform on Cys and Lys microenvironments. These results implicate Lys residue proximity as a factor when considering alcohol metabolism's impact on Cys redox status, specifically when a Lys is within 5–7.5 Å (Fig. 4C). Indeed, 95 % of redox-modified Cys have at least one proximal or within 15 Å Lys residue. Interestingly, this trend did not apply to Lys acetylation, given that 47 % of acetyl-Lys are within 15 Å of a Cys residue and 53 % are farther than 15 Å from a Cys residue (Fig. 4C).

### 3.4 Cys-Lys proximity motifs

**Fig. 5:**
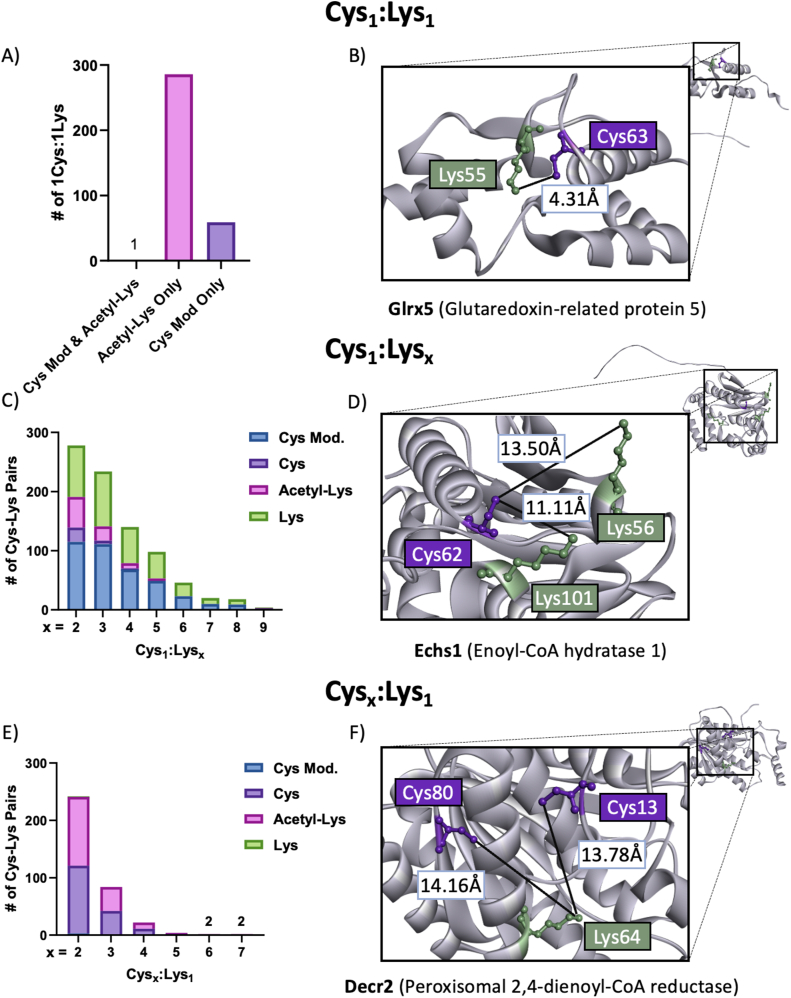
CysLys pair motifs. A) Cys_1_:Lys_1_. Number of pairs with redox changed or unchanged Cys paired with an acetylated or unmodified Lys. B) AlphaFold2 predicted structure of Glrx5 which contains one Cys-Lys pair with a redox modified cysteine and acetylated Lys. Cys-Lys pairs: Cys63 – Lys55. C) Cys_1_:Lys_x_. Bar plot showing the distribution of pairs comprised of a Cys residue proximal to multiple Lys residues, with x representing the number of Lys residues. D) AlphaFold2 predicted structure of Echs1 contains a singular cysteine residue proximal to three lysine residues. Cys-Lys pairs: Cys62 – Lys101, Lys56. E) Cys_x_:Lys_1_. Bar plot showing the distribution of pairs comprised of a Lys residue proximal to multiple Cys residues, with x representing the number of Cys residues. F) AlphaFold2 predicted structure of Decr2 which contains two cysteine residues proximal to a singular lysine residue. Cys-Lys pairs: Lys62 – Cys13, Cys80. Cys: Cysteine; Lys: Lysine.

Our integrated proteome analyses revealed four different motifs of Cys-Lys pairs modified by alcohol consumption. These include proteins containing: 1) one Lys proximal to one Cys (Cys_1_:Lys_1_); 2) one Lys proximal to multiple Cys (Cys_x_:Lys_1_); 3) one Cys proximal to multiple Lys (Cys_1_:Lys_x_); and 4) multiple Lys proximal to multiple Cys (Cys_x_:Lys_x_). The pairs evaluated contained at least one residue with an alcohol-induced PTM (i.e., redox-modified Cys and/or acetylated Lys residue) to ensure a mechanistic interpretation of alcohol-induced biochemical pathologies (Supplemental 3 CysLys Motif). Additionally, enrichment analysis was conducted on the proteins containing different Cys-Lys motifs. The different motifs are enriched for varying subcellular locations, biological processes, and enzymatic classes (Table 1). The Cys_1_:Lys_1_ motif predominately contains acetylated Lys with 99 % unmodified Cys within 15 Å (Fig. 5A). In total, 345 Cys_1_:Lys_1_ pairs were identified and Glrx5 was selected to demonstrate the 1Cys:1Lys relationship (Fig. 5B). The most common of these motifs was Cys_1_:Lys_x_ with 419 Cys-Lys pairs quantified in total. With the Cys_1_:Lys_x_ motif, the majority of Cys were found to have undergone a significant change in redox status (Fig. 5C) and this is illustrated with Echs1 (Fig. 5D). Evaluation of Cys_x_:Lys_1_ motifs showed that all Lys but 1 within this motif were acetylated/deacetylated out of 178 Cys-Lys pairs (Fig. 5E). Decr2 was used to illustrate the Cys_x_:Lys_1_ motif (Fig. 5F). Finally, an example of the Cys_x_:Lys_x_ motif is detailed in the next section using the alcohol metabolizing enzyme, Adh1.

### 3.5 Evaluation of Adh1 binding sites containing Cys-Lys pairs

**Fig. 6:**
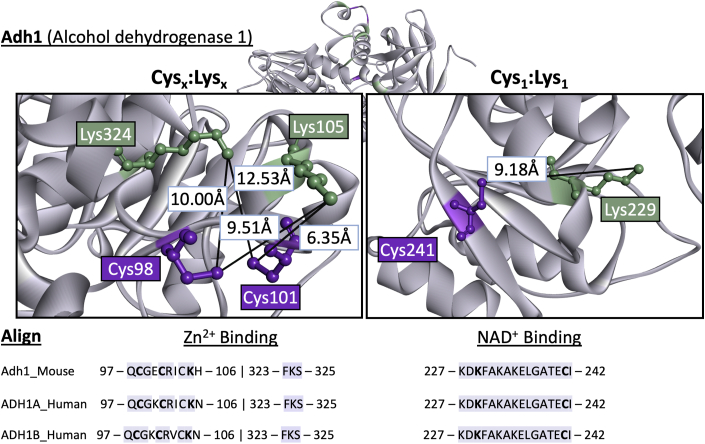
CysLys pairs are conserved within the zinc and NAD^+^ binding sites of Adh1. The panel to the left depicts cysteines that coordinate Adh1 zinc and the proximal lysine residues. The right panel depicts the Lys that binds to NAD^+^ and its proximal cysteine. Adh1 structure was generated by AlphaFold2. Amino acid sequences between mouse and human Adh1 were aligned. Bolded within the Zn^2+^ and NAD^+^ binding region represent the amino acids involved in Zn^2+^ binding and NAD^+^ binding. NAD^+^: Nicotinamide adenine dinucleotide; Zn^2+^: Zinc.

Adh1 is a primary ethanol metabolizing enzyme in the liver which drives the reduction in the NAD+/NADH ratio and acetaldehyde (AcH) generation. This enzyme included both a Cys_1_:Lys_1_ pair and the Cys_x_:Lys_x_ motif in different locations of the protein. At the NAD ^+^ binding pocket a Cys_1_:Lys_1_ pair was identified and the Lys229 residue was found to be a part of NAD ^+^ binding [30]. Furthermore, Lys229 (FC = 1.66, p (Corr) = 0.01) was found to be significantly acetylated proximal to Cys241. In contrast, the zinc binding pocket was found to have a Cys_x_:Lys_x_ motif with three of the zinc coordinating Cys to be significantly reduced by alcohol (Cys98, FC = 4.95, p (Corr) = 0.01; Cys101 FC = 8.27, p (Corr) = 0.01) and are proximal to two acetylated Lys (Lys324 FC = 1.99, p (Corr) = 0.03; Lys105 FC = 1.27, p (Corr) = 0.05) (Fig. 6) [30,31]. Alignment of human ADH1 isoforms and mouse Adh1 amino acid sequences revealed that Cys-Lys pairs within zinc and NAD^+^ binding sites were 100 % conserved between species and isoforms (Fig. 6) [30].

### 3.6 Identification of human proteins orthologous to murine proteins with Cys-Lys pairs detected within an acetylomic profile of severe alcohol-associated liver hepatitis explants

**Fig. 7:**
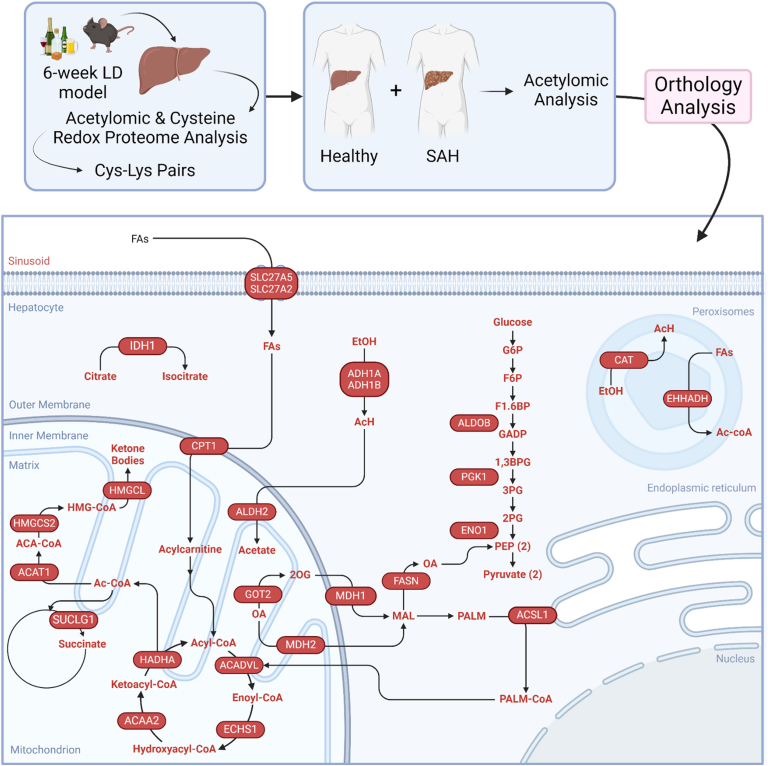
Proteins identified to be deacetylated within severe alcohol-associated hepatitis (SAH) explant tissue containing Cys-Lys pairs separated by subcellular compartments and associated pathways. Depicted are orthologous enzymes of murine proteins containing Cys-Lys pairs and human proteins found deacetylated within SAH explant tissues. All enzymes are represented by red ovals and the substrates they interact with. The pathways that were enriched are placed within their respective subcellular location and include glycolysis/gluconeogenesis, alcohol metabolism, fatty acid metabolism, citric acid cycle and ketogenesis. Figure made in BioRender.com. ACADVL: Very long-chain specific acyl-CoA dehydrogenase; ACA-CoA: Acetoacetyl-CoA; Ac-CoA: Acetyl-CoA; ACAT1: Acetyl-CoA acetyltransferase 1; ACSL1: Acetyl-CoA synthetase 1; SAH: severe alcohol-associated hepatitis; ALDOB: Aldolase fructose-bisphosphate B; CBR1: Carbonyl reductase 1; EHHADH: Enoyl-CoA hydratase and 3-hydroxyacyl CoA dehydrogenase; ENO1: Enolase 1; FAs: Fatty acids, FASN: Fatty acid synthase; HADHA: Hydroxyacyl-CoA dehydrogenase trifunctional protein; HMGCL: 3-hydroxy-3-methylglutaryl-CoA lyase; HMGCS2: Hydroxymethylglutaryl-CoA synthase; GOT2: Glutamic-oxaloacetic transaminase 2; IDH1: Isocitrate dehydrogenase 1; LD: Lieber-DeCarli; MDH1: Malate dehydrogenase 1; MDH2: Malate dehydrogenase 1; PALM-CoA: Palmitoyl-CoA; PEP: Phosphoenolpyruvate; PGK1: Phosphoglycerate kinase 1; SUCLG1: Succinate-CoA ligase.

Characterizing how alcohol metabolism impacts protein biochemistry through undiscovered mechanisms is key to understanding the pathological progression of alcohol-associated end-stage liver disease and therapeutic development. Therefore, translating our murine findings to the human condition is paramount. Thus, the Ensembl genome browser was used to identify orthologous proteins containing one or more Cys-Lys pair modified by alcohol metabolism (see Supplemental 4 SAH CysLys Orthology and Enrichment). The human orthologous proteins identified were cross referenced with an acetylomic analysis conducted on hepatic explants from SAH patients [27]. The human protein orthologs with murine counterparts containing Cys-Lys pairs modified by alcohol metabolism were all found to have significantly decreased acetylation in the patients with SAH. Thus, all hepatic proteins with altered acetylation in patients with SAH were found to have Cys-Lys pairs in our murine model of ALD. To assess how Cys-Lys orthologs translate to disease pathology, the list of proteins was submitted to DAVID, identifying significantly enriched biological processes. Then Reactome was used to determine enzyme substrate use and connection within biological processes (Fig. 7). The processes enriched included ethanol metabolism, glycolysis/gluconeogenesis, β-oxidation, TCA and ketogenesis (see Supplemental 4 SAH CysLys Orthology and Enrichment). Key enzymes within these pathways such as ADH1A/B, aldolase B (ALDOB), ECHS1, succinate-CoA ligase (SUCLG1), and hydroxyacyl-CoA dehydrogenase trifunctional protein (HADHA) were identified, offering avenues for exploring how these PTMs impact protein structure and function in the context of disease pathology (Fig. 7).

## 4 Discussion

Alcohol metabolism alters the hepatic proteome through both a reduction in the Cys proteome and Lys hyperacetylation. Here, we show for the first time that these alcohol-induced protein modifications may regulate protein structure and redox function due to Cys-Lys proximity. Indeed, both Cys and Lys are independently regulated through a host of well-characterized PTMs such as Cys-glutathionylation or Lys-ubiquitination [32]. However, this newly discovered feedback mechanism plays a key role across a host of metabolic processes by uniquely sensing 1) the hepatic metabolic state by acetylation and 2) oxidative stress via thiol redox signaling. Indeed, this coordinated crosstalk among Cys-Lys pairs highlights key redox signaling hubs responsible for hepatic responses to alcohol toxicity.

Our whole liver acetylomic analysis of murine tissue revealed that many acetylated proteins were within the ETC (Fig. 2). These findings suggest that the acetylation of the ETC complexes results in diminished respiratory output which is supported by a decrease in ATP and NAD^+^ consequent to alcohol metabolism [33,34]. Additionally, several of the sites found to be acetylated by alcohol consumption have been previously identified in Sirt3-deficient mice including NADH:ubiquinone oxidoreductase (Complex I) and ATP synthase (Complex V) [35,36]. This directly correlates with the pathway analysis enrichment of the sirtuin signaling pathway noted above. Sirt3 is the major deacetylase in the mitochondria, which is the subcellular compartment with the most acetylated proteins, making Sirt3 vital to regulating acetylation in ALD [37]. Sirt3-deficient murine models have decreased ATP concentrations, supporting the concept that Lys hyperacetylation results in ETC inhibition [10]. These findings have been validated within ATP synthase and succinate dehydrogenase (SDHA, Complex II). For example, ATP synthase acetylation decreases protein activity and is regulated by Sirt3 [36]. Also, hyperacetylation of Complex II blocks substrate entry, inhibits activity, and is also regulated by Sirt3 [24,38]. Furthermore, Complex I acetylation has been found to wholly control respiratory output within other models and is likely a crucial component to NAD^+^ depletion in ALD [39,40]. Overall, the evaluation of alcohol-induced whole liver acetylation and the interrogation of the cross talk between the hepatic acetylomic and cysteine proteomic alterations revealed a critical interplay of carbon and redox stress.

The integration of proteins found by the acetylomic and redox proteomic analyses revealed an alcohol-related association between redox active Cys residues and Lys acetylation. The proteins identified across these datasets were primarily comprised of oxidoreductase enzymes or enzymes that catalyze redox reactions (Fig. 3B), and the mitochondria and cytosol are the most common subcellular locations of these proteins (Fig. 3C). Among the Toxicity Functions identified by IPA, liver steatosis was one of the most enriched, validating our murine model of early-stage ALD (Fig. 3D). Proteins associated with liver steatosis are central to β-oxidation, for example, acyl-CoA synthetase long chain family member 1 (ACSL1) breaks down long chain fatty acids. Similarly, carnitine palmitoyltransferase I (CPT1A) transports fatty acids into the mitochondria. Two other proteins were also found related to β-oxidation, HADHA and acyl-CoA dehydrogenase (ACADS) [41,42]. Each of these enzymes are critical in the transport, oxidation, and metabolism of fatty acids, revealing that Cys redox signaling and Lys acetylation play a role in alcohol-induced redox signaling. Canonical pathway analysis found both modifications to be central to the regulation of lipid and xenobiotic metabolism through enzymes regulated by the RXRA transcription factor within the liver (Fig. 3E). Changes in RXRA concentrations have been connected to IL-1, which are proinflammatory cytokines well documented to be elevated within ALD patients and contribute to hepatocyte disfunction leading to fibrosis/cirrhosis [43]. Subsequently, IL-1 and alcohol consumption diminish RXRA concentrations resulting in the disruption of lipid handling [44]. Studies have identified an association between depleted RXR protein levels and the inhibition of fatty acid oxidation leading to hepatic lipid accumulation in ALD [45]. Integrating our datasets to identify key protein signatures through Cys and Lys modification enabled the identification of new mechanisms of metabolic control essential to lipid handling and the pathogenesis of fat deposition in the early stages of ALD.

The evaluation of 3D protein structures and quantifying the proximity between redox-modified Cys and Lys residues revealed specific paired Cys and Lys sites involved in enzymatic regulation. Furthermore, this analysis demonstrated that Cys redox status was more responsive to Lys proximity. In our model of ALD, the optimal distance for a Lys to impact Cys reduction appears to be 5-7.5 Å (Fig. 4C). The strong structural link between alcohol metabolism and the effect it had on the Cys proteome is exemplified by the finding that 95 % of redox-modified Cys residues were paired with a proximal Lys (Fig. 4C). The mechanism driving this interaction is yet to be elucidated but is likely due, in part, to the reductive stress generated during the metabolism of alcohol to AcH and acetate. Interestingly, the proximity of a Cys only resulted in Lys acetylation 50.5 % of the time. This suggests that Lys acetylation is occurring through additional mechanisms such as other structural protein features or environmental factors. The alkaline pH of the mitochondria and elevated concentrations of Ac-CoA are both noted as an evolutionary mechanism resulting in fewer Cys-Lys pairs conserved versus the cytosol, specifically in longer living species, such as non-human primates and humans [46]. Contrary to longer lived species, mice have conserved Cys-Lys pairs within their mitochondrial matrix [13]. The metabolic feedback loop driven by the generation of Ac-CoA and increased likelihood of enzymatic inhibition has not evolutionarily impacted Cys-Lys pair conservation in mice. This necessitates cross referencing murine Cys-Lys pairs and conservation within the human proteome. An important evolution of our dataset is the finding that proximal Cys-Lys pairs regulate Cys reactivity with the potential for alcohol-induced Lys acetylation to impact protein function outside the canonical functions of Lys modification.

Embedded within the alcohol-induced proximal Cys-Lys findings is the potential for protein regulation. Our analysis revealed that unique Cys-Lys structural motifs may define enzymatic processes. These motifs describe shared amino acid spatial interactions directed through electrostatics to facilitate the transfer of an acetyl moiety from a Cys thiol to a Lys ε-amine, thus impacting Cys reactivity. The four motifs identified are separated by the number of Cys and Lys residues within a defined 15 Å sphere and are noted in Table 1 above and illustrated in Fig. 5, Fig. 6. One of the proteins containing a Cys_1_:Lys_1_ pair was Glrx5, found to have been reduced at Cys63 (FC = 3.27, p (Corr) = 0.026) and paired with acetyl Lys55 (Fig. 5B). This impacts a regulatory hotspot as Lys55 binds to glutathione and Cys63 coordinates the 2Fe–2S cluster [47]. The reduction of a redox active Cys indicates diminished enzymatic activity resulting from alcohol metabolism. Another example of this motif is glutamate oxaloacetate transaminase 2 (Got2), which is acetylated at Lys404 (FC = 2.66, p (Corr) = 0.0002) and is proximal to Cys382. The acetylation of Got2 at Lys159, Lys185 and Lys404 has been associated with an increased affinity for MDH2 (Malate dehydrogenase 2) and is regulated by Sirt3 [48]. This provides a clear example of a proximal Cys-Lys pair involved in the redox regulation that has been conserved as a regulatory mechanism to control enzyme activity during sustained oxidative stress. Illustrating the second motif, Echs1 contains a singular Cys proximal to multiple Lys (Cys_1_:Lys_x_) and was found to be modified at Lys101 and Cys62 (FC = 3.94, p (Corr) = 0.002 and FC = 5.01, p (Corr) = 0.01, respectively) (Fig. 5D). Cys62 is additionally proximal to Lys101, Lys56, and Lys115. Lys101 contributes to enoyl-CoA substrate binding and acetylation at Lys101 is proposed to inhibit enzymatic activity [49]. ECHS1 deficiency is associated with alterations in metabolic activity including diminished oxidative phosphorylation and dysregulation of fatty acid oxidation, both commonly observed in models of ALD [50,51]. A third motif is characterized by having multiple Cys paired with one proximal Lys. Decr2 contains this Cys_x_:Lys_1_ motif where Lys64 is proximal to Cys13 and Cys80. Lys64 aids in NADP ^+^ binding and was significantly deacetylated by alcohol metabolism (FC = −1.53, p (Corr) = 0.01) [5,52]. Decr2 carries out the degradation of peroxisomal unsaturated fatty enoyl-CoA esters and this proximal Cys-Lys pair likely plays an important role in lipid homeostasis. Importantly, this site was previously found to be acetylated in a model of ALD [5,53]. The proximity of multiple Cys residues to a Lys in this motif could signify that these conserved Cys-Lys pairs induce S → N transfers resulting in the acetylation of these sites and regulating protein activity. The final motif identified is Cys_x_:Lys_x_ which was found within Adh1, a key enzyme in alcohol pathogenesis. Adh1 was further evaluated due to the presence of Cys-Lys motifs (Cys_x_:Lys_x_ and Cys_1_:Lys_1_) within the zinc and NAD^+^ binding regions (Fig. 6). Adh1 catalyzes the reduction of alcohol to AcH using zinc to transfer electrons, reducing NAD^+^ to NADH [30]. Our redox proteomic analysis revealed that Cys residues involved in the coordination of zinc were reduced, suggesting that zinc was no longer bound. This is further supported by the identification of acetylated proximal Lys which is likely acetylated via S → N acetyl moiety transfer. As stated above, Lys229 (FC = 1.66, p (Corr) = 0.01) a component of the NAD^+^ binding site was found to be acetylated while also being proximal to Cys241 [30]. The inhibition of Adh1 could be one mechanism to limit the amount of AcH generated and to conserve hepatic NAD^+^ concentrations. Supporting this idea, previous researchers have shown that Adh1 inhibition is protective, decreasing lipogenic genes and triglyceride accumulation in mice exposed to alcohol [9]. Indeed, AcH inhibits the transport of NADH into the mitochondria, limiting cofactor concentrations. Understanding how these four Cys-Lys motifs regulate activity must be carried out on an individual protein basis and will be key to revealing potential therapeutic targets focused on the early intervention of ALD. Future studies may also require the analysis of protein quaternary structures, such as dimer and tetramer multimers, since there is a high chance that Cys-Lys pairs exist throughout these proteoforms.

Our analysis reveals that alcohol-induced metabolic disturbances leave their mark on Cys-Lys pairs in a unique manner. Our murine hepatic Cys-Lys analysis aligned translationally when compared with acetylation targets identified previously in SAH human explants (Fig. 7). SAH is a complex disease that damages hepatocytes and impairs metabolic activity resulting in decreased acetylation across protein lysine residues and provides a unique signature that inversely aligns with murine acetylation in an early model of ALD, where fully functioning hepatocytes are actively metabolizing alcohol [27]. This alignment suggests that Cys-Lys motifs correlate in a regulatory nature across species and murine Cys-Lys pairs are comparable to the human condition. Several alcohol metabolizing orthologs were identified by our spatial Cys-Lys analysis. Two of the human ADH1 isoforms, ADH1A and ADH1B, as well as Catalase (CAT) and Aldehyde dehydrogenase (ALDH2) were found to have decreased acetylation in the SAH hepatic explants and align with our murine Cys-Lys pairs [54]. In addition to ethanol metabolizing enzymes, the TCA cycle and glycolysis/gluconeogenesis were found to contain similar proximal Cys-Lys pairs in SAH tissue, with altered acetylation profiles [55]. Glycolytic enzymes found to be deacetylated in SAH explants with orthologous murine Cys-Lys pair(s) include ALDOB, Phosphoglycerate kinase 1 (PGK1) and Enolase 1 (ENO1). Inhibition of ETC, TCA, and glycolysis contribute to a metabolic shift toward fatty acid synthesis and steatosis. Consequently, there would be an increase in lipid uptake by fatty acid transporters of the solute carrier family (SLC), SLC27A5 which was found to be significantly reduced and SLC27A2 which was found to be significantly reduced and acetylated in mice. Also, Acetyl-CoA synthetase 1 (ACSL1), Very long-chain specific acyl-CoA dehydrogenase (ACADVL), and HADHA were found to be significantly reduced and acetylated likely contributing to impaired lipid catabolism due to our murine model of ALD [56].

Notably, numerous ketogenic enzymes were found to contain proximal Cys-Lys pairs within our murine and SAH acetylomic analysis, including CPT1, Acetyl-CoA acetyltransferase 1 (ACAT1), MDH2, Hydroxymethylglutaryl-CoA synthase (HMGCS2) and 3-hydroxy-3-methylglutaryl-CoA lyase (HMGCL). CPT1 is critical to initiating ketogenesis by transporting long-chain fatty acids into the mitochondria and converting them into fatty acyl-carnitines. This results in increased Ac-CoA concentrations via β-oxidation and elevated acetoacetyl-CoA (ACA-CoA) and thus initiating the ketogenic pathway [8,57]. Additionally, this pathway is induced when oxaloacetate levels are low as with the activation of MDH2 via acetylation and reducing oxaloacetate to malate while oxidizing NADH to NAD^+^ [48]. HMGCS2 will then use ACA-CoA to generate β-hydroxy β-methylglutaryl-CoA (HMG-CoA). Furthermore, HMGCS2 was found to be acetylated at regulatory Lys310 in the absence of SIRT3, potentially impacting the generation of ketone bodies [58]. Further evaluation of these pathways is critical to understanding protein specific changes that could contribute to the metabolic disruption induced by alcohol consumption. Vital targets bridge models of ALD and SAH pathology solidifying the necessity of multiple approach analysis for therapeutic target identification.

Overall, our analysis reveals that alcohol metabolism specifically modifies Cys-Lys redox-centric protein motifs and supports the hypothesis that these unique metabolic-sensing thiol signaling hubs play a key role in the pathogenesis of 10.13039/100026725ALD. Future work will continue to characterize spatially resolved Cys-Lys biochemical mechanisms in protein pathways associated with the onset and progression of ALD with a focus on lysine acetylation, redox modifications, and impact on enzyme function. These results begin to define the complex spatiotemporal relationships protein modifications play in directing adaptive responses to altered hepatic redox signaling resulting from alcohol consumption.

## CRediT authorship contribution statement

**Courtney D. McGinnis:** Writing – review & editing, Writing – original draft, Visualization, Validation, Software, Methodology, Investigation, Formal analysis, Data curation. **Peter S. Harris:** Writing – review & editing, Visualization, Validation, Methodology, Investigation, Formal analysis, Data curation. **Brenton I.M. Graham:** Validation, Software, Formal analysis. **John O. Marentette:** Methodology, Investigation, Data curation. **Cole R. Michel:** Writing – review & editing, Validation, Methodology, Investigation, Formal analysis, Data curation. **Laura M. Saba:** Writing – review & editing, Visualization, Validation, Software, Investigation, Funding acquisition, Formal analysis. **Richard Reisdorph:** Writing – review & editing, Validation, Methodology, Formal analysis, Conceptualization. **James R. Roede:** Writing – review & editing, Writing – original draft, Validation, Methodology, Investigation, Conceptualization. **Kristofer S. Fritz:** Writing – review & editing, Writing – original draft, Visualization, Validation, Supervision, Software, Resources, Project administration, Methodology, Investigation, Funding acquisition, Formal analysis, Data curation, Conceptualization.

## Declaration of competing interest

The authors declare no conflicts of interest.

## Data Availability

Data will be made available on request.

## Appendix A Supplementary data

Multimedia component 1 Supplemental 1 Cys Lys StructureMap. Multimedia component 1

Multimedia component 2 Supplemental 2 IPA Acetylome and CysLys. Multimedia component 2

Multimedia component 3 Supplemental 3 CysLys Motif. Multimedia component 3

Multimedia component 4 Supplemental 4 SAH CysLys Orthology and Enrichment. Multimedia component 4

Multimedia component 5 See Supplemental Word document for Supplemental Tables 1–3. Multimedia component 5

The mass spectrometry proteomics dataset and the whole liver acetylomics dataset are available via the ProteomeXchange Consortium at the PRIDE partner repository using the database identifier PXD054686 (http://www.ebi.ac.uk/pride/archive/). The cysteine redox proteomic dataset is available via ProteomeXchange Consortium with the dataset identifier PXD042070.
