# Supplementary material for: Acetylation of proximal cysteine-lysine pairs by alcohol metabolism
Source: Redox Biol. 2024 Dec 12;79:103462. doi: 10.1016/j.redox.2024.103462 (PMC11732177; doi:10.1016/j.redox.2024.103462)

**Supplemental Table 1.**Comparison of the odds of a redox-modified cysteine in proteins with an acetylated lysine to the odds of a redox-modified cysteine in proteins without an acetylated lysine (605 proteins).

| Cysteine Proteome | Acetyl-Lys | No Acetyl-Lys | Odds Ratio (Fisher’s exact test) |
| --- | --- | --- | --- |
| Modified | 227 | 192 | 1.47 (p-value=0.034) |
| Not Modified | 83 | 103 |  |

**Supplemental Table 2.** Comparison of the odds of an acetylated lysine in proteins with a redox-modified cysteine to the odds of an acetylated lysine in proteins without a redox-modified cysteine (1,005 proteins).

| Acetylome | Redox Cys | No Redox Cys | Odds Ratio (Fisher’s exact test) |
| --- | --- | --- | --- |
| Modified | 214 | 435 | 1.33 (p-value=0.054) |
| Not Modified | 96 | 260 |  |

**Supplemental Table 3.**Comparison of the odds of an acetylated lysine in Cys-Lys pairs (within 15 Å of each other) with a redox-modified cysteine to the odds of an acetylated lysine in Cys-Lys pairs without a redox-modified cysteine (28,238 Cys-Lys pairs).

| Acetylome | Redox Cys | No Redox Cys | Odds Ratio (Fisher’s exact test) |
| --- | --- | --- | --- |
| Modified | 211 (9%) | 1,295 (5%) | 1.88 (p-value<0.0001) |
| Not Modified | 2,135 (91%) | 24,597 (95%) |  |

**Supplemental Figure 1. Immunoblot evaluation of whole liver acetylation.**

A) Pan-acetylation immunoblot demonstrates a greater number of proteins that contain acetylated lysines in the liver tissue of mice consuming alcohol.

B) Quantitation for the immunoblot, after normalization with trichloroethanol total protein, represented as the percent of the control with standard error of the means. Showing a 1.9-fold increase of acetylated lysines due to ethanol consumption. (n=5 per group, ***p <0.001).

Supplemental Figure 1


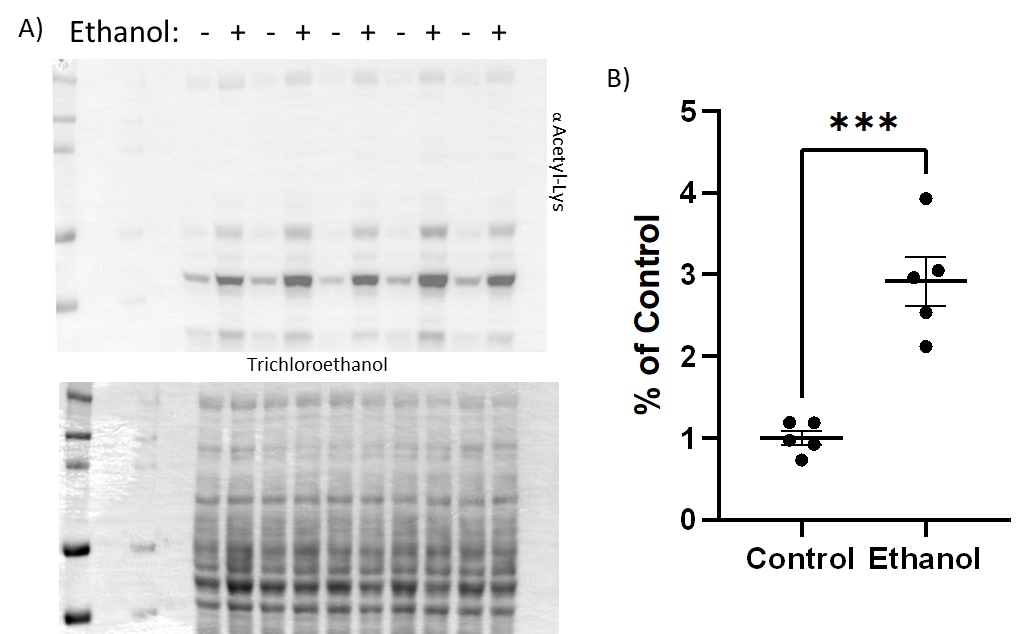

Supplement: Multimedia component 5 — See Supplemental Word document for Supplemental Tables 1–3. [file mmc5.docx]
